# Growth Responses of Wheat (Triticum aestivumL. var. HD 2329) Exposed to Ambient Air Pollution under Varying Fertility Regimes

**DOI:** 10.1100/tsw.2003.64

**Published:** 2003-08-20

**Authors:** Anoop Singh, S. B. Agrawal, Dheeraj Rathore

**Affiliations:** Lab of Air Pollution and Global Climatic Change, Department of Botany, Allahabad Agricultural Institute- Deemed University, Allahabad- 211 007, India

**Keywords:** air pollution, fertility level, plant growth, total biomass, yield, Triticum aestivum

## Abstract

The problem of urban air pollution has attracted special attention in India due to a tremendous increase in the urban population; motor vehicles vis a vis the extent of energy utilization. Field studies were conducted on wheat crops (Triticum aestivum L. var. HD 2329) by keeping the pot-grown plants in similar edaphic conditions at nine different sites in Allahabad City to quantify the effects of ambient air pollution levels on selected growth and yield parameters. Air quality monitoring was done at all the sites for gaseous pollutants viz. SO_2_, NO_2_, and O_3_. Various growth parameters (plant height, biomass, leaf area, NPP, etc.) showed adverse effects at sites receiving higher pollution load. Reduction in test weight and harvest index was found to be directly correlated with the levels of pollutant concentrations. The study clearly showed the negative impact of air pollution on periurban agriculture.

## INTRODUCTION

Rapid industrialization, especially in urban areas of India, is affecting the air quality[1]. Cities in developing countries tend to have high vehicle densities and therefore are likely to experience a high contribution from motor vehicles to the total urban pollution load. In developing countries, vehicle fleets tend to be older and poorly maintained, a factor that increases the significance of motor vehicles as a pollutant source[2]. Another cause of air pollution is emissions from various heavy and medium industries in and around cities, which produce deleterious effects on human health, economic plants, and general vegetation. The data from urban cities in India, using a range of different analytical methods, have demonstrated a 7-h mean $O_3$ concentration in summer months, which exceeds 40 ppb and in some cases approaches 60 ppb[3].

Crop production is highly dependent on environmental conditions, among which air quality plays a major role. Urban air pollution has a direct impact on periurban agriculture due to dispersion of emissions in all directions along the wind. During transportation, primary pollutants often form secondary pollutants causing greater adverse effects in periurban areas. Air pollutants ($O_3$, $SO_2$, etc.) in ambient air have long been known to be phytotoxic[4,5] and cause the greatest amount of any gaseous pollutants by reducing growth and productivity of many plant species through reduction in photosynthesis, accelerated leaf senescence, and decreased root growth[6,7].

The nature and extent of impact of a mixture of pollutants on vegetation may not be the same as that of a single pollutant. The response depends on the type of species, nature and intensity of the pollutant, duration of exposure, and interaction with other environmental factors[8,9]. Ayer and Bedi[10] recorded highest degradation in growth performance, biochemical parameters, and yield of wheat in the most polluted zone of Baroda City. Wahid et al.[11] have demonstrated a grain yield reduction of 46 and 38% for two cultivars of wheat in an open top chamber study in the vicinity of Lahore (Pakistan) using ambient and charcoal filtered air, respectively.

Mineral nutrient supply may increase pollutant injury to crops[12], but some studies have indicated that plants grown at low nutrient supply are more sensitive to air pollutant injury[13,14,15]. Ormrod and Adedipe[16] have suggested that mineral nutrients may modify the responses of plants to air pollutants and responses vary with the specific element and species under consideration.

While the impact of air pollutants on agriculture in developed countries has received considerable attention, there has been little recognition of its potential impacts in developing countries, including India. Emissions of major air pollutants are growing rapidly in different parts of the country, with industrialization, urbanization, and the growth of transport, while the high temperature and high solar radiation are proving favorable for production of high concentrations of $O_3$.

In view of the above, the present study was undertaken to assess the impact of air pollution on growth and yield of wheat (*Triticum aestivum* L. var. HD 2329) in periurban areas of Allahabad City.

## MATERIALS AND METHODS

The study was performed in the periurban and rural environment of Allahabad City, Eastern Gangetic plains of India at 24° 47’ N latitude, 81° 19’ and 82° 21’ E longitudes, and 315’ above mean sea level. The climate of Allahabad City is tropical monsoonic with three distinct seasons, i.e., summer, rainy, and winter. Annual average temperature is 24°C, RH 65%, and 959 mm annual precipitation. The traffic on highways is dominated by heavy commercial vehicles, while in other areas there is multiplicity of vehicles along with nonmotorized vehicles. In many places, there is a disruption in the free flow of traffic due to narrow and poorly maintained roads, so increasing the emission of pollutants. The plant species *Triticum aestivum* L. chosen for this study is a staple food for India.

Wheat (*Triticum aestivum* L. var. HD 2329) plants were grown from germination to maturity in pots having similar edaphic conditions at nine different sites viz. Allahabad Agricultural Institute (AAI), Arail (Ar), Sadwakala (Sd), Bahrana (Bh), Jhunsi (Jh), Prayag (Pr), Mehdeori (Mh), Civil lines (CL), and Rajrooppur (RRP) around and within the city, under seven treatments of fertilizers (i.e., without fertilizer [$F_0$]; recommended dose of N, P, and K [$F_1$]; one and half times of recommended dose of N, P, and K [$F_2$]; two times of recommended dose of N, P, and K [$F_3$]; recommended dose of N and P [$F_4$]; recommended dose of P and K [$F_5$]; and recommended dose of N and K [$F_6$]). Nitrogen, phosphorous, and potassium were given in the form of urea, single super phosphate, and murate of potash, respectively. A half dose of nitrogen and full dose of phosphorous and potassium was given as basal dressing and another half as top dressing. Pots were placed in an unshaded area and received uniform light as measured by a light intensity meter. Plants were watered twice a week and received 500 ml $pot^{-1}$ deionized water in each watering. For analysis, triplicate random samples of plants from each treatment of each site were taken. For total biomass determination, plants were oven dried at 80°C until the constant weight was obtained and values were expressed as g $plant^{-1}$.

Portable gas samplers performed air quality monitoring weekly, for 6 h daily (10 AM to 4 PM) for $SO_2$, $NO_2$, and $O_3$ using wet chemical methods. $SO_2$, $NO_2$, and $O_3$ were scrubbed separately in tetra chloromurcurate, NaOH (0.1N) and buffered KI (0.1N), respectively. These absorbing solutions were later analyzed colorimetrically for $SO_2$[17], $NO_2$[18], and $O_3$[19] pollutants. No continuous advanced gas monitors are available in Allahabad and this wet chemical sampling regime was the maximum possible with the resources available. Monitoring of pollutants for more than 6 h is also not possible due to the safety of samplers during the night *vis a vis* failure/availability of electricity at various sites as samplers have battery backup of only 6 h.

## RESULTS

Results of air monitoring are shown in Table 1, which clearly indicated that RRP was the most polluted site among all the selected experimental sites and Sd was least polluted. The concentrations of $SO_2$, $NO_2$, and $O_3$ were recorded in the range between 30.83 to 42.50, 38.13 to 65.04, and 17.0 to 30.83 µg m-3, respectively at RRP, and at Sd (reference site) these gases were found in the range between 2.5 to 10.0, 10.23 to 14.55, and 5.5 to 12.92 µg m-3, respectively.

**TABLE 1**
**Concentrations of $SO_2$, $NO_2$ and $O_3$ (µg $m^{-3}$ air) at Different Sites During Experiment**

| Experimental Sites | $SO_2$ | | $NO_2$ | | $O_3$ | |
|---|---|---|---|---|---|---|
| | Min. | Max. | Min. | Max. | Min. | Max. |
| RRP | 30.83 | 42.5 | 38.13 | 65.04 | 17.0 | 30.83 |
| Ar | 2.5 | 12.5 | 10.16 | 18.29 | 3.33 | 13.33 |
| AAI | 15.83 | 30.0 | 14.23 | 36.26 | 10.66 | 28.33 |
| Sd | 2.5 | 10.0 | 10.23 | 14.55 | 5.5 | 12.92 |
| CL | 10.0 | 17.5 | 14.23 | 24.22 | 10.0 | 25.0 |
| Jh | 2.5 | 22.5 | 10.13 | 22.36 | 8.33 | 17.66 |
| Mh | 2.5 | 17.5 | 14.23 | 18.13 | 7.08 | 24.17 |
| Pr | 12.5 | 17.5 | 15.48 | 22.36 | 10.42 | 28.75 |
| Bh | 25.0 | 37.5 | 14.39 | 46.09 | 12.91 | 29.59 |

Reduction in plant height and biomass accumulation was recorded due to increase in levels of air pollution depending on the site (Figs. 1, 2). $F_2$ treatment showed positive impact by increasing these parameters at all the sites. Plant height and total biomass were recorded maximum 66.13 cm and 3.74 g, respectively, at Sd in $F_2$ treatment. At all sites, $F_5$ treatment (without N) was found less effective to overcome the losses through air pollution, in comparison to $F_4$ and $F_6$ (without K and P, respectively). In $F_4$, $F_5$, and $F_6$, plant height and biomass were recorded 50.87, 40.97,

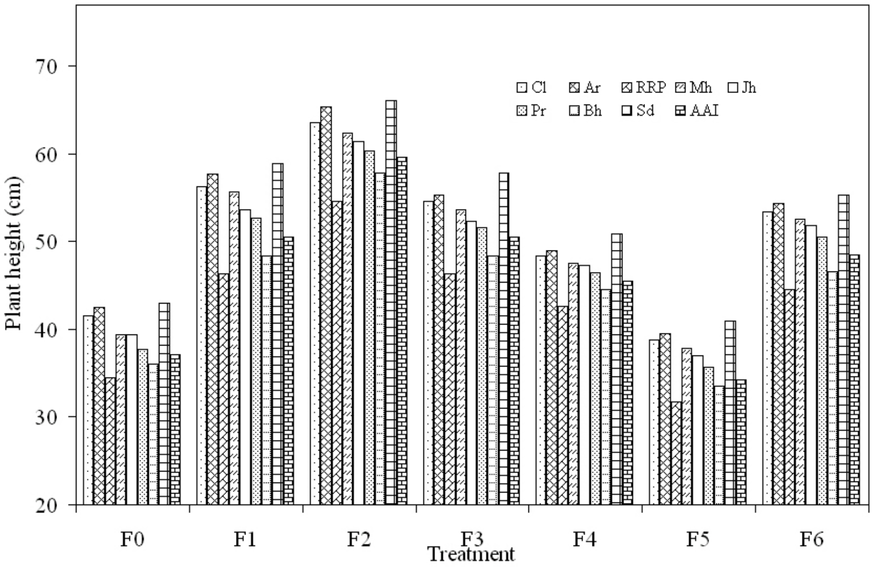

**FIGURE 1.** Effect of different fertility levels on plant height of wheat plants grown at various sites.

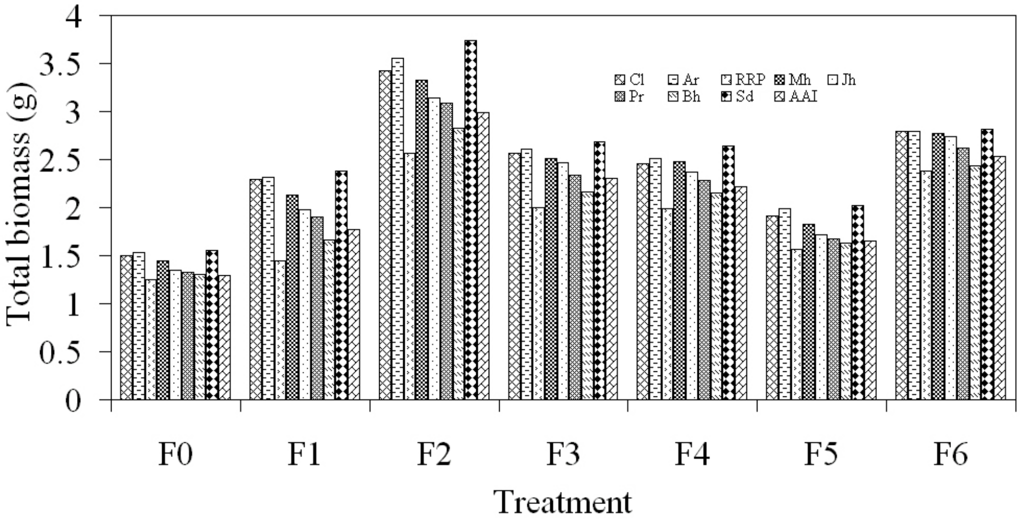

**FIGURE 2.** Effect of different fertility levels on total biomass of wheat plants grown at various sites.

and 55.30 cm and 2.613, 2.022, and 2.82 g, respectively. Results of two-way ANOVA showed significant variation ($p < 0.001$) in total biomass due to sites and treatment (Table 2). Leaf area and net primary productivity (NPP) were also recorded maximum in $F_2$ treatment at all experimental sites (Figs. 3, 4). Leaf area increased from 30.41 cm$^2$ ($F_0$ treatment) to 62.25 cm$^2$ ($F_2$ treatment) at RRP. Maximum NPP was observed in $F_2$ treatment at each experimental site and recorded to be maximal (0.0357 g plant$^{-1}$ day$^{-1}$) at Sd. Among $F_4$, $F_5$, and $F_6$, minimum increase in leaf area (18.91%) was observed in $F_5$ treatment (without N) while $F_4$ and $F_6$ showed 59.19 and 75.63% increase with respect to $F_0$ (control) at Sd. Two-way ANOVA test showed significant effect ($p < 0.001$) of treatments and level of pollutant concentration on NPP (Table 2).

**TABLE 2**
**Variance Ratio for Growth and Yield Parameters of Wheat Plants Grown at Different Fertility Levels**

| Parameters | Site | Treatment | Site x Treatment |
|---|---|---|---|
| Plant height | *** | *** | NS |
| Total biomass | *** | *** | *** |
| Leaf area | *** | *** | NS |
| NPP | *** | *** | *** |
| Test weight | *** | *** | NS |
| Harvest index | ** | *** | *** |

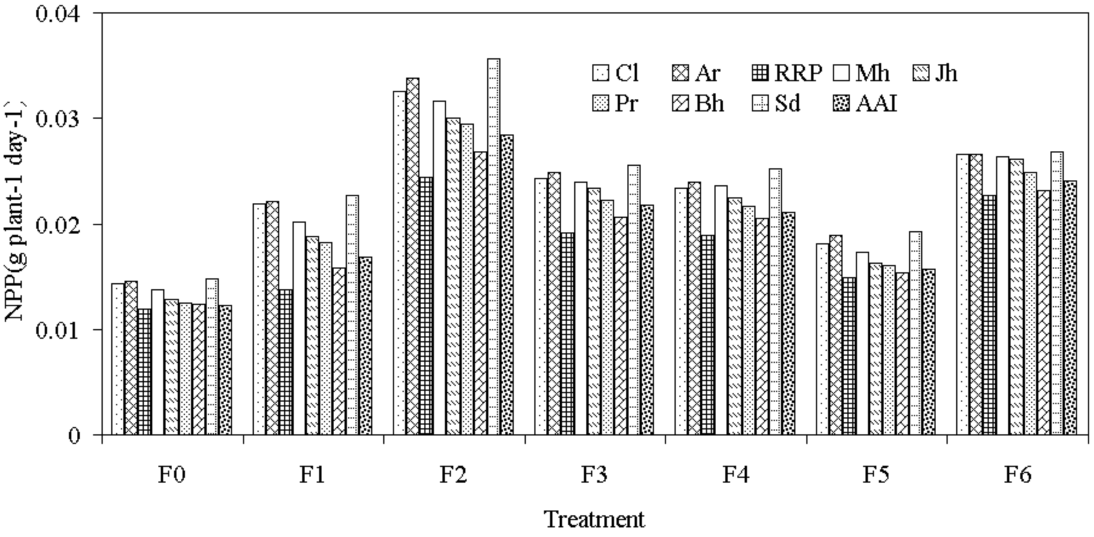

**FIGURE 3.** Effect of different fertility levels on net primary productivity of wheat plants grown at various sites.

Test weight (1000 seed weight) of wheat plants grown at various sites significantly decreased with increasing pollution load (Table 3). Minimum test weight (20.45 g) was recorded at RRP in $F_0$ treatment, which increased up to 28.52 g in $F_2$. Maximum test weight (34.30 g) was found at Sd in $F_2$ treatment. Increase in test weight was less in $F_5$ treatment (0.39%) than $F_4$ and $F_6$ (29.29 and 13.79%, respectively) at RRP. Harvest index (HI) has also shown similar results, and it decreased with increasing levels of air pollutants (Fig. 5). $F_2$ treatment minimized the pollutant-induced adverse effect by increasing the test weight and harvest index of wheat plants. HI increased up to 18.74% ($F_2$) from 11.73% ($F_0$), at RRP. Losses in yield were recorded maximum at highly polluted sites (Fig. 6). Yield losses were recorded to be 10.24 to 23.0% at Sd used as a reference site (showing minimum pollution load) in $F_0$ treatment, which reduced up to 2.08 to 14.81% in $F_2$ treatment, while $F_1$ showed maximum yield loss, i.e., 21.29% at RRP and minimum (5.05%) at Ar. Among $F_4$, $F_5$, and $F_6$, maximum yield loss (22.91%) was found in $F_5$ treatment, while 16.41 and 18.15% yield losses were recorded in $F_4$ and $F_6$, respectively, at RRP.

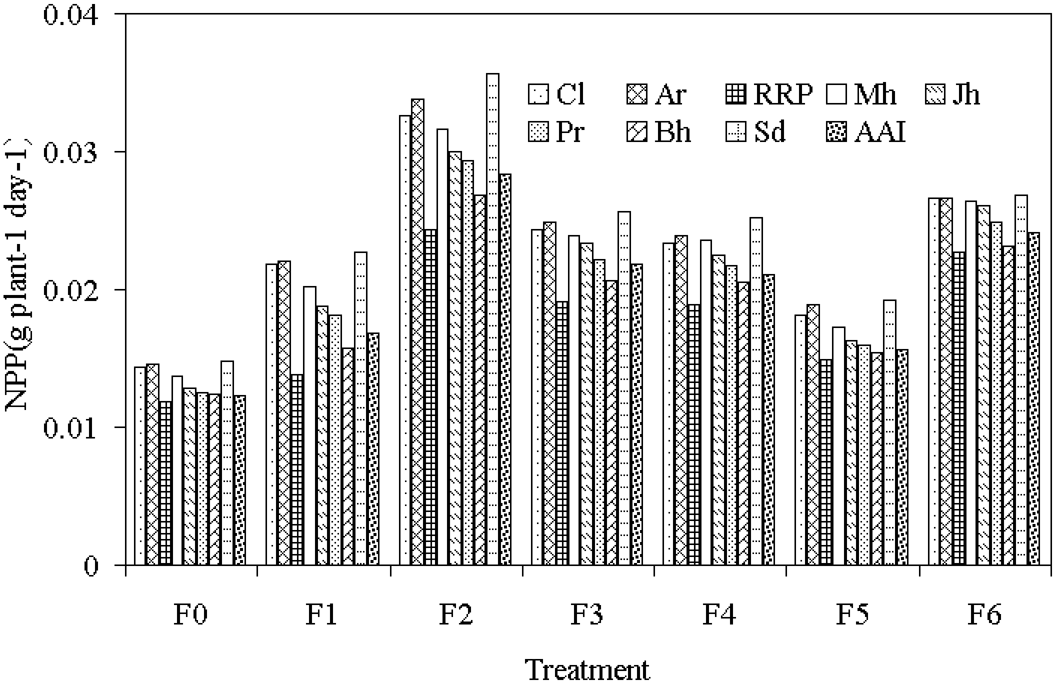

**FIGURE 4.** Effect of different fertility levels on leaf area of wheat plants grown at various sites.

**TABLE 3**
**Effect of Different Fertility Levels on Test Weight (g) of Wheat Plants Grown at Various Sites***

| Sites | Treatments | | | | | | |
|---|---|---|---|---|---|---|---|
| | $F_0$ | $F_1$ | $F_2$ | $F_3$ | $F_4$ | $F_5$ | $F_6$ |
| RRP | $20.45 \pm 0.37^{d}$ | $25.61 \pm 0.80^{b}$ | $28.52 \pm 0.47^{a}$ | $26.42 \pm 0.39^{b}$ | $26.44 \pm 0.38^{b}$ | $20.53 \pm 0.51^{d}$ | $23.27 \pm 0.23^{c}$ |
| Ar | $25.65 \pm 0.10^{d}$ | $31.89 \pm 0.67^{b}$ | $33.60 \pm 0.36^{a}$ | $30.67 \pm 0.39^{b}$ | $30.70 \pm 0.27^{b}$ | $26.17 \pm 0.53^{d}$ | $27.73 \pm 0.35^{c}$ |
| AAI | $21.74 \pm 0.45^{d}$ | $27.57 \pm 0.49^{b}$ | $30.58 \pm 0.67^{a}$ | $28.48 \pm 0.88^{ab}$ | $28.88 \pm 0.53^{ab}$ | $23.60 \pm 0.69^{cd}$ | $24.49 \pm 1.03^{c}$ |
| Sd | $26.24 \pm 0.26^{e}$ | $32.53 \pm 0.46^{b}$ | $34.30 \pm 0.51^{a}$ | $31.20 \pm 0.24^{c}$ | $31.63 \pm 0.47^{bc}$ | $26.63 \pm 0.39^{e}$ | $28.43 \pm 0.44^{d}$ |
| CL | $24.82 \pm 0.40^{d}$ | $30.84 \pm 0.37^{b}$ | $32.73 \pm 0.42^{a}$ | $30.23 \pm 0.31^{b}$ | $30.57 \pm 0.77^{b}$ | $25.60 \pm 0.40^{d}$ | $27.67 \pm 0.63^{c}$ |
| Jh | $22.32 \pm 0.67^{e}$ | $28.60 \pm 0.51^{b}$ | $31.68 \pm 0.47^{a}$ | $29.39 \pm 0.54^{b}$ | $29.42 \pm 0.53^{b}$ | $24.75 \pm 0.64^{d}$ | $26.63 \pm 0.84^{c}$ |
| Mh | $24.41 \pm 0.32^{d}$ | $29.63 \pm 0.44^{b}$ | $32.47 \pm 0.84^{a}$ | $29.46 \pm 0.31^{b}$ | $30.07 \pm 0.59^{b}$ | $25.42 \pm 0.51^{cd}$ | $26.67 \pm 0.69^{c}$ |
| Pr | $22.20 \pm 0.58^{d}$ | $28.34 \pm 0.48^{b}$ | $31.39 \pm 0.56^{a}$ | $28.66 \pm 0.44^{b}$ | $29.65 \pm 0.68^{ab}$ | $24.70 \pm 0.80^{c}$ | $25.49 \pm 0.54^{c}$ |
| Bh | $21.53 \pm 0.52^{d}$ | $26.21 \pm 0.59^{b}$ | $29.35 \pm 0.46^{a}$ | $27.57 \pm 0.78^{ab}$ | $27.33 \pm 0.64^{b}$ | $22.35 \pm 0.61^{d}$ | $24.26 \pm 0.67^{c}$ |

- Mean ± 1SE, value followed by the same letter within a column is not significantly different ($p < 0.05$), using Duncan's Multiple Range test.

## DISCUSSION

Air pollution can influence plant species in diverse ways and thus affect ecosystems at various levels of organization[20]. A positive correlation was observed among the pollutants (viz. $SO_2$,

$NO_2$, and $O_3$). In another experiment, the $O_3$ concentration was negatively correlated to nitric oxide (NO), but was positively correlated to ($NO_2$), moreover, an $O_3$ level and $NO_2$/NO[21]. Agrawal[22] reported that the annual NOx concentrations varied from 10 to 90 µg $m^{-3}$ in various parts of the country. Varshney and Agrawal[23] have shown ground level $O_3$ concentration between 20 to 273µg $m^{-3}$ in Delhi. Back ground concentrations of $O_3$ have been reported to increase during the last decades and it is expected that they follow rising next years [24, 25].

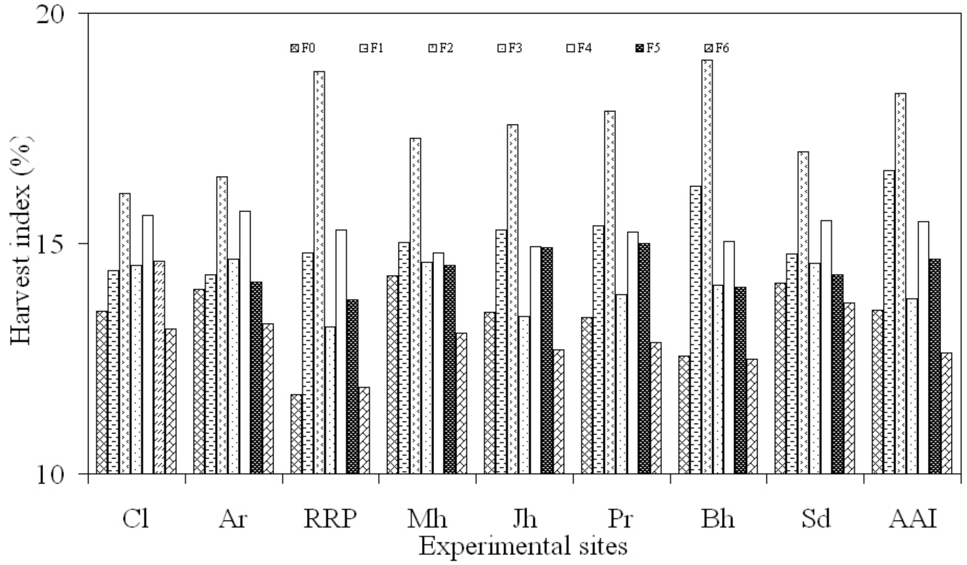

**FIGURE 5**. Effect of different fertility levels on harvest index of wheat plants grown at various sites.

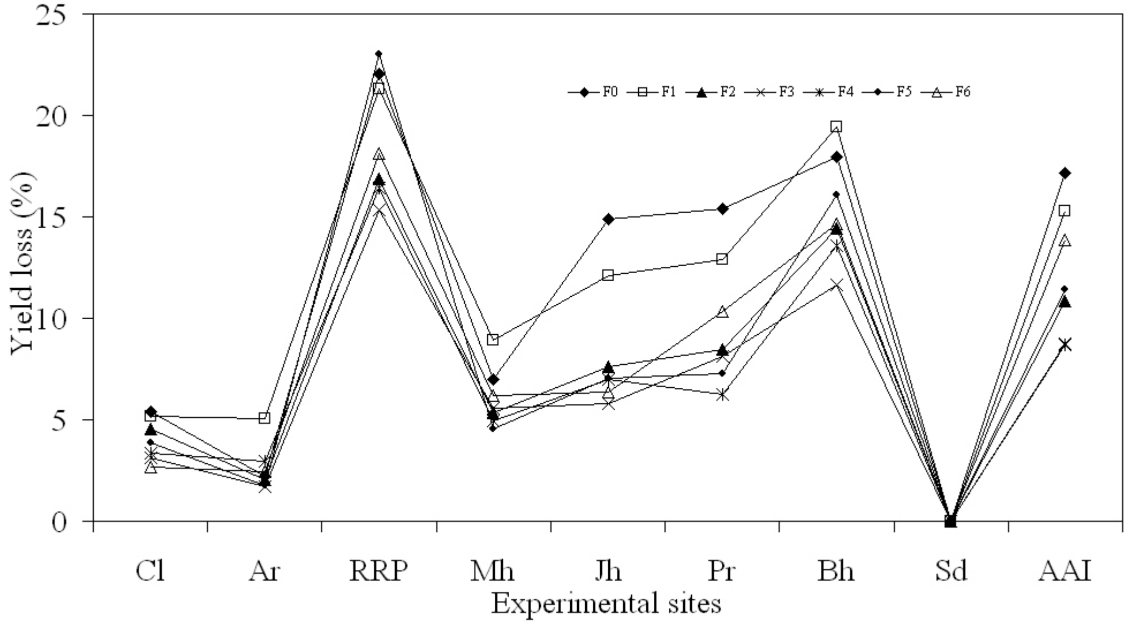

**FIGURE 6.** Effect of different fertility levels on yield loss against Sd (reference site) of wheat plants grown at various sites.

The deterioration in air quality has been shown to adversely affect the crop growth. Reduction in plant height and total biomass accumulation was observed, in the present study, with an increase in pollution load. Pandey and Agrawal[26] have reported reductions in height of three woody perennials under varying air pollution stress in the urban environment of Varanasi, the adjoining city of Allahabad. Ashmore et al.[27] have also reported a decline in biomass accumulation in different plant parts along a gradient of air pollution around London. Any detrimental acidification due to the products of $SO_2$ and $NO_x$ pollution may consequently have an inhibitory effect on processes such as $CO_2$ fixation[28]. Soil nutrition has a dramatic effect on the sensitivity of vegetation to air pollutants. Increase in biomass accumulation in the radish plant was observed by Kostka-Rick[29] with increasing nitrogen supply and was reduced due to a long-term chronic exposure of air pollutants. Agrawal and Verma[30] reported that total plant height was reduced significantly in $SO_2$ treated plants, except those grown using recommended and twice the recommended N, P, and K applications.

Ziska and Caulfield[31] also noticed greater $O_3$ damage in ragweed plants due to continuous exposure until maturity. The joint action of $O_3$ and $SO_2$ caused significant suppression in dry matter of tomato shoot and root at all concentrations[32]. Rao and DeKok[33] and Verma et al.[15] reported reduction in biomass accumulation in wheat plants due to higher levels of $SO_2$. Shahare and Varshney[34] observed that plant height, number of branches, nodes, and leaf per plant were reduced due to $SO_2$ exposure. McKee et al.[35] reported that elevated $O_3$ caused a 15% decline in total biomass accumulation in wheat plants.

$F_2$ treatment showed positive impact on plant height and biomass by decreasing the negative impact of air pollutants. Verma et al.[15] suggested that nutrient status modifies the response of wheat cultivars to $SO_2$. Accumulation of dry matter was higher in fertilizer-amended plants as compared to the unamended ones. Nutrient application has stimulated plant growth and a large proportion of absorbed sulfur due to $SO_2$ exposure is being utilized in metabolic processes rather than accumulated as $SO_4^{-2}$[15]. Nitrogen supply also increases leaf photosynthesis via the amount of N-containing component such as ribulose-1, 5 bisphosphate carboxylase/oxygenase activity[36].

Leaf area and NPP were negatively affected with increasing pollution load. Topa et al.[37] also found significant reduction in biomass and leaf area of sugar maple seedling due to $O_3$. Since biomass accumulation is a function of net carbon gain, it is not surprising that reported declines in growth resulting from $O_3$ are often correlated with reduction in photosynthesis[38]. Krupa et al.[39] also suggested that atmospheric pollution by $O_3$ could result in depression in plant biomass and crop yield. Such a depression reflects a decline in carbon gain, caused by an inhibition of photosynthesis that could be mainly attributed to reduced carboxylation activity of Rubisco and/or to a decrease in Rubisco quantity[40]. $O_3$, $SO_2$, and $NO_2$ individually and in combination are known to reduce the yield of many crop plants[41,42]. Plants supplemented with NPK fertilizers were less damaged by $SO_2$ pollution[14,43,44]. NPP is highly correlated with biomass accumulation. In the present investigation, reduction in biomass was observed at heavier polluted sites, resulting that NPP also decreased. Significant reductions in leaf area and biomass of potato plants were also reported by Petitte and Ormrod[45] due to $SO_2$ and $NO_2$ treatment. Ambient $O_3$ concentrations in Europe can cause a range of effects including visible leaf injury, growth and yield reductions, and altered sensitivity to biotic and abiotic stresses[46,47]. Ayer and Bedi[10] reported maximum damage in various growth parameters viz. root length, shoot length, leaf area, biomass, NPP, RGR, and grain yield in *Triticum aestivum* plants due to urban industrial air pollution in Baroda City (India). However, Ziska[48] suggested that $O_3$ levels associated with urban environment in the U.S. might not limit the growth or reproductive development of ragweed. According to Ollerenshaw and Lyons[49] the reduction in grain yield in field grown winter wheat induced by $O_3$ was due to decrease in number of grains per spikelet or due to the increases in number of infertile florets. It is well documented that N and P deficiency reduces chlorophyll concentration[50,51]. Nutrient deficiency has modified the carbon allocation pattern in plants exposed to $SO_2$ while nutrient amendment has lowered the magnitude of reduction in chlorophyll and also photosynthesis[30]. Stephens[52] found enhanced growth of young *Nothofagus fusca* tree by increasing N supply.

The supply of N, P, and K has increased the yield of wheat by increasing photosynthetic activity in foliar tissue, which have further reduced the magnitude of reduction in biomass and yield due to air pollutants. Coleman et al.[53] have suggested that plants growing in nutrient-poor conditions may be more sensitive to air pollution with respect to changes in carbon gain. N limitation has been shown to decrease chlorophyll and protein content, RuBP carboxylase activity, and increase of mesophyll resistance, which all limit $CO_2$ fixation[54]. High P availability is found to increase the rate of photosynthesis[50]. K fertilization is also beneficial due to its role in stomatal opening, photosynthesis, protein synthesis, osmotic regulation, and pH regulations[55]. The increase in yield may be attributed to the favorable effect of nutrients on photosynthesis, biomass accumulation and consequently on translocation of assimilates to reproductive parts.

## CONCLUSION

In the present study, the wheat plants were maintained under similar climatic and edaphic conditions but with varying air pollution load and observed the differences in plant growth performance. As pollutant concentrations increased, the plant height, total biomass, leaf area, NPP, and RGR decreased *vis a vis* decline in test weight and HI indicating that air pollutants suppressed the growth and yield of wheat plants. It might be due to reduction in physiological and biochemical processes and restrain of defense mechanism. Enhanced fertilizer than the recommended dose ($F_2$) resulted in the positive response by increasing the total biomass, test weight, and total yield. Response of individual nutrients showed that N was most important than P and K. Potassium was least important, to overcome the negative impact of air pollutants.

The present investigation suggests that urban air quality of Allahabad is affecting the agriculture production unfavorably in periurban areas. Further research is required in this direction on some important crop plants to increase the food production to meet the requirement of growing population of developing countries like India.

## ACKNOWLEDGMENTS

The authors wish to express sincere thanks to Prof. R.B. Lal, Vice Chancellor, Allahabad Agricultural Institute (DU) and Prof. P.W. Ramteke, Director (Research) for providing laboratory facilities and encouragement. Authors are also grateful to HRD Division of C.S.I.R, Govt. of India (New Delhi) for providing financial support in the form of a research project.
